# Shaping hemophilia care: lessons and legacy of the SIPPET trial after 10 years

**DOI:** 10.1016/j.rpth.2026.106649

**Published:** 2026-05-08

**Authors:** Flora Peyvandi, Roberta Palla, Isabella Garagiola, Pier Mannuccio Mannucci

**Affiliations:** 1 Department of Pathophysiology and Transplantation, Università degli Studi di Milano, Milan, Italy; 2 Fondazione Istituto di Ricovero e Cura a Carattere Scientifico Ca’ Granda Ospedale Maggiore Policlinico, Angelo Bianchi Bonomi Hemophilia and Thrombosis Center, Milan, Italy

**Keywords:** factor VIII, genetics, hemophilia A, incidence, von Willebrand factor

## Abstract

The advent of nonreplacement therapies, such as emicizumab, has contributed to a marked reduction of the observed incidence of factor (F)VIII inhibitors in patients with severe hemophilia A. However, it should be clarified whether this decline reflects delayed FVIII exposure or a true reduction in immunogenicity. Thus, understanding the mechanisms driving anti-FVIII inhibitor formation, particularly during early exposure in previously untreated patients, remains a key objective in hemophilia care and is a still unmet need. Ten years ago, the Survey of Inhibitors in Plasma-Product Exposed Toddlers (SIPPET) trial marked a turning point by providing the first randomized evidence that plasma-derived FVIII products containing von Willebrand factor are associated with a lower inhibitor incidence than recombinant FVIII. These findings influenced international guidelines, prompted changes in clinical practice, and highlighted the importance of the choice of the product type during the early, immunologically vulnerable period of exposure to FVIII. Post-SIPPET studies expanded this evidence base, offering deeper insights into the immunogenicity of FVIII products and offering mechanistic insights into the influence on inhibitor development of gene mutations, nonneutralizing antibodies, immunoglobulin G subclass responses, epitope specificity, and epigenetic modulation. The legacy of SIPPET is not only in its immediate clinical impact but also in its catalyzing role for a broader, multidisciplinary effort to understand inhibitor occurrence in severe hemophilia A.

## Essentials

• Survey of Inhibitors in Plasma-Product Exposed Toddlers (SIPPET) compared the inhibitor risk of plasma-derived vs recombinant factor (F)VIII in infants.
• The trial evaluated randomly early FVIII exposure in previously untreated patients to assess immune outcomes.
• Mechanistic works showed how FVIII-immune interactions initiate inhibitor development.
• SIPPET findings shaped global guidance and early therapy choices to limit inhibitor incidence.

## 1 Introduction

Hemophilia A (HA) is an X-linked congenital bleeding disorder caused by mutations in the *F8* gene, resulting in deficiency or dysfunction of coagulation factor (F)VIII [1]. The phenotypic severity of HA is typically classified according to FVIII activity plasma levels: severe (<1 IU/dL), moderate (1-5 IU/dL), and mild (>5-40 IU/dL) [2]. Severe HA is associated with spontaneous bleeding episodes, particularly in joints and muscles, which lead to chronic arthropathy and disability if left untreated [3]. The mainstay of treatment is replacement therapy with FVIII concentrates, which can be either plasma-derived (pdFVIII) containing von Willebrand factor (VWF) or produced by recombinant DNA technology (rFVIII) [3]. The most severe complication of replacement therapy is the development of neutralizing anti-FVIII antibodies, commonly referred to as inhibitors [4,5]. These alloantibodies bind to infused FVIII, rendering it ineffective and leading to increased morbidity, treatment complexity, and healthcare costs.

Inhibitor development is a multifactorial event, influenced by genetic factors such as the type of *F8* mutation, family history of inhibitor, and immune-related gene polymorphisms, as well as by environmental and treatment-related factors including the type of FVIII product, intensity, and timing of first exposure to FVIII plus concomitant infections or inflammatory stimuli [6]. Among previously untreated patients (PUPs) with severe HA, at least 30% develop inhibitors, high-titer inhibitors (≥5 Bethesda units) occurring in approximately half of them [7]. The high incidence for this complication of therapy underscores the clinical relevance of identifying risk factors and strategies for primary prevention. Recent data from the European Haemophilia Safety Surveillance (EUHASS) registry demonstrate a significant reduction of inhibitors after the introduction of the nonreplacement product emicizumab as first-line prophylaxis of bleeding [8]. In 2016, the cumulative incidence of inhibitors in severe HA was 24%, but by 2022, incidence was reported to be 6% at a time when 44% of PUPs with severe HA received emicizumab for prophylaxis. This does not mean that there is a true reduction in immunogenicity. The use of emicizumab in PUPs significantly diminishes onset, frequency, and intensity of FVIII exposure, so that the decline of inhibitor incidence may be due to this new treatment paradigm, delaying the inhibitor development. However, as a scientific community we must not lose focus on the main unanswered question: “what does drive inhibitor development?”

The Survey of Inhibitors in Plasma-Product Exposed Toddlers (SIPPET) study was the first randomized controlled trial directly comparing the immunogenicity of pdFVIII and rFVIII in PUPs with severe HA [9]. This trial and subsequent analyses have contributed to our understanding of FVIII immunogenicity, not only revealing differences in inhibitor risk associated with product type and early time of exposure but also highlighting the importance of patient-specific risk stratification. Moreover, a number of post-SIPPET studies explored the role in shaping the immune responses to FVIII of nonneutralizing antibodies, immunoglobulin G (IgG) subclass distribution, FVIII epitope recognition, and epigenetic modifications. The purpose of this review was to provide a synthesis of the evidence stemming from SIPPET and related studies.

## 2 The Sippet Trial: Design, Outcomes, and Early Lessons

The SIPPET trial randomized 251 PUPs with severe HA in a 1:1 ratio to first receive pdFVIII containing VWF or rFVIII products [9]. Participants were followed for 50 consecutive exposure days, 3 years, or until inhibitor development. PUPs were enrolled across 42 multinational treatment centers. Inhibitors developed in 29 of 125 cases treated with pdFVIII (20 with high-titer inhibitors) and in 47 of 126 cases treated with rFVIII (30 with high-titer inhibitors). The cumulative incidence of all inhibitors was 26.8% for pdFVIII, 44.5% for rFVIII, and 18.6% and 28.4% for high-titer inhibitors. Cox regression models indicated that rFVIII was associated with an 87% higher inhibitor risk than pdFVIII (hazard ratio [HR], 1.87; 95% CI, 1.17–2.96). Except for the type of *F8* mutation, no clear associations were observed between inhibitor risk and previously evaluated factors such as race/ethnicity, treatment intensity and age at first treatment. Thus, 10 years ago, SIPPET first provided compelling evidence for a differential immunogenicity between pdFVIII and rFVIII products.

In addition, a post hoc SIPPET analysis evaluated the temporal pattern of inhibitor onset. Inhibitors peaked within the first 10 exposure days (EDs), with the highest contrast between rFVIII and pdFVIII observed within the first 5 EDs [10]. For rFVIII, HRs for the initial 5 EDs were 3.14 for all inhibitors and 4.19 for high-titer inhibitors. In contrast, the peak for pdFVIII occurred later (6-10 EDs). This temporal vulnerability underscores the heightened immunologic risk during early FVIII exposure and has implications for early treatment planning.

## 3 Real-World Impact: Translating Sippet Findings Into Clinical Practice

The SIPPET trial findings stimulated extensive debate and prompted reevaluation of clinical practice and treatment strategies in the frame of the markedly evolving therapeutic landscape of HA in the last 10 years.

### 3.1 Controversies, debates, and real-world evidence

While SIPPET provided grade I evidence, criticisms emerged regarding the generalizability of the findings, exclusion of novel nonfactor therapies, and that rFVIII products were not fully represented in SIPPET following the advent of extended plasma half-life (EHL) products [[11], [12], [13], [14]]. A central question emerged: is the increased inhibitor risk with rFVIII observed in a multinational randomized trial confirmed in real-world observational cohorts? Several large registries and national studies have since provided valuable insights. The FranceCoag retrospective cohort examined 395 PUPs treated between 2000 and 2010. Calvez et al. [15] reported that the cumulative inhibitors incidence was higher in cases treated with rFVIII than with a pdFVIII product, particularly in the early exposure days. These results closely mirrored the SIPPET findings, reinforcing the signal that rFVIII is more immunogenic (Table 1) [9,[15], [16], [17], [18]]. The EUHASS and the Canadian Bleeding Disorders Registry have tracked for 11 and 8 years inhibitor onset in 1219 PUPs with severe HA after 50 exposure days. Results showed a lower inhibitor incidence with pdFVIII (20%; 95% CI, 14%-26%) than with standard half-life rFVIII (27%; 95% CI, 24%-30%), yielding an odds ratio of 0.67 (95% CI, 0.45-0.98; *P* = .04) [18] (Table 1).

**Table 1:** Chronology and characteristics of observational and randomized studies comparing inhibitor development in PUPs with hemophilia A receiving pdFVIII or rFVIII.

Study name	Type of study	Total patients (*N*)	No. PUPs with rFVIII products	No. PUPs with pdFVIII products	Type of rFVIII products	Type of pdFVIII products	Cumulative incidence			Adjusted RR/adjusted HR/OR
							Overall	rFVIII products	pdFVIII products	
CANAL [16]	Multicenter observational cohort study	316	181	135	• Kogenate • Helixate • Kogenate Bayer • Recombinate • ReFacto	24 different pdFVIII^a^	26% (82/316)^b^	29% (53/181)^b^	21% (29/135)^b^	RR, 0.7; 95% CI, 0.4-1.1
RODIN [17]	Multicenter observational cohort study	574	486	88	• Recombinate • Kogenate • Helixate • ReFacto • Kogenate FS • Helixate NexGen/FS • ReFacto AF • Advate	15 different pdFVIII^c^	32.4% (95% CI, 28.5-36.3)	32.3%	33.1%	HR, 0.96; 95% CI, 0.62-1.49 HR, 1.60; 95% CI, 1.08-2.37 for second-generation full-length recombinant products
SIPPET [9]	Multicenter randomized cohort study	251	126	125	• Recombinate • Kogenate FS • Advate • ReFacto AF	• Alphanate • Fanhdi • Emoclot • Factane	35.4% (95% CI, 28.9-41.9)	44.5% (95% CI, 34.7-54.3)	26.8% (95% CI, 18.4-35.2)	HR, 1.87; 95% CI, 1.17-2.96
FranceCoag cohort [15]	Multicenter observational cohort study	395	264	131	• Advate • Kogenate	Factane	35.0%^d^ (95% CI:30.2%-40.3%)	31.6% Advate^d^ (95% CI, 23.9-41.1) 50.1% Kogenate^d^ (95% CI, 41.6-59.4)	22.5%^d^ (95% CI, 15.8-31.5)	HR, 1.41 (0.83-2.38) for Advate vs Factane HR, 2.74 (1.67-4.4838) for Kogenate vs Factane
EUHASS and CBDR cohort [18]	Multicenter observational cohort study	1219	1031	188	• Advate • Kogenate • Helixate NexGen • Recombinate • ReFacto • ReFacto AF/Xyntha • Kovaltry • Novoeight • Nuwiq	• Beriate • Emoclot • Factane • Fanhdi • Haemoctin SDH • Immunate • Octanate • Haemate P • Hemophil M • Voncento • Wilate	25.6% (95% CI, 23.2%-28.1%)	26.9% (95% CI, 24.2%-29.8%)	19.7% (95% CI, 14.3%-26.1%)	OR, 0.67; 95% CI, 0.45%-0.98%; *P* = .04

CANAL, Concerted Action on Neutralizing Antibodies in severe hemophilia; CBDR, Canadian Bleeding Disorders Registry; EDs, exposure days; EUHASS, European Haemophilia Safety Surveillance; HR, hazard ratio; NA, not available; OR, odds ratio; pdFVIII, plasma-derived factor VIII; PUP, previously untreated patient; rFVIII, recombinant factor VIII; RODIN, Research Of Determinants of INhibitor development among previously untreated patients with severe hemophilia; RR, relative risk; SIPPET, Survey of Inhibitors in Plasma-Product Exposed Toddlers.

a Aafact; Alphanate; α-Profilate; Amofil; Beriate; BPL 8Y; Criostat; Emoclot; FVIII SD; Fanhdi; factor VIII LFB; fresh frozen plasma; Haemate P; Hemofil-M; Hemofil T; Hemoctin; Immunate; Innovate; Koate; Monoclate; Octonativ M; Replenate; Uman-Cry; and FVIII product from a local blood bank (Dutch).

b Inhibitor incidence.

c Aafact; Amofil; Beriate; cryoprecipitate; Emoclot; FVIII-LFB; Fanhdi; fresh frozen plasma; FVIII Intersero; Haemate P; Haemoctin; Hemofil; Immunate; Octanate; and Wilate.

d Global cumulative incidence at 75 EDs.

In this context, it must be appreciated that concerns regarding the immunogenicity of rFVIII products date back to the early 1990s, because it was suspected that differences with pdFVIII regarding posttranslational processing and tertiary structure would promote neoantigen formation. In early studies involving PUPs, rFVIII products showed a high cumulative incidence of inhibitors (approximately 25%–30%) [19,20]. Subsequently an array of observational studies and a systematic review showed also a much higher inhibitor risk in PUPs treated with rFVIII than with pdFVIII, reporting a more than 2-fold risk increase [4,21].

At variance, 2 large observational, retrospective studies, Concerted Action on Neutralizing Antibodies in severe hemophilia A (CANAL) [16] and Concerted Action on Neutralizing Antibodies in severe hemophilia A (RODIN) study [17], included 574 PUPs and found no difference in inhibitor risk between pdFVIII and rFVIII (Table 1). Further results from RODIN also revealed a divergent immune response among recombinant products. The so-called second-generation products were associated with a higher inhibitor risk than third-generation products (adjusted HR, 1.60; 95% CI, 1.08-2.37) (Table 1). Subsequent reports confirmed the higher immunogenicity of rFVIII products endowed with a standard plasma half-life in the frame of several independent cohorts, including cases enrolled in the Réseau FranceCoag [22] and those registered in the United Kingdom Haemophilia Centre Doctors' Organisation National Haemophilia Database [23]. In addition, meta-analyses and systematic reviews that incorporated data stemming from observational studies reported a higher inhibitor risk with rFVIII than with pdFVIII [[24], [25], [26]], but the difference was attenuated after adjustment for potential confounders. More recently, observational studies that evaluated in PUPs standard or EHL rFVIII products showed that the overall inhibitor incidence ranged approximately 30% or more (Table 2) [[27], [28], [29], [30], [31], [32]]. A lower incidence of inhibitors was reported for simoctocog alfa, an rFVIII product produced in human cell lines in contrast to those evaluated in SIPPET, all manufactured in hamster cell lines [27]. Pertaining to EHL products, interim results from the phase 3 study of rurioctocog alfa pegol suggested a lower inhibitor incidence. However, the prevalence of high-risk *F8* gene variants and a family history of FVIII inhibitors were lower in this cohort compared with previously published studies including SIPPET, suggesting that the enrolled patients had a lower baseline risk of inhibitor development [31].

**Table 2:** Characteristics and inhibitor incidence of observational studies carried out in PUPs with hemophilia A treated exclusively with standard and extended plasma half-life recombinant FVIII.

Study name	Type of study	Product name (brand name)	Type of rFVIII product	Type of FVIII molecule	Total PUPs (*N*)	Overall inhibitor incidence	High-titer inhibitor incidence
NuProtect Study [27]	Prospective, multinational, open-label, noncontrolled, phase 3 study (NCT01712438)	Simoctocog alfa (Nuwiq)	Standard half-life rFVIII produced by human cell	Full-length FVIII	105	26.7% (28/105)	16.2% (17/105)
Guardian 4 study [28]	Multicenter, multinational, nonrandomized, open-label phase 3 trial (NCT01493778)	Turoctocog alfa (Novoeight)	Standard half-life rFVIII	BDD-FVIII	58	43.1% (25/58)	27.6% (16/58)
AFFINITY extension study [29]	Phase 3, open-label, multicenter, extension study (NCT02172950)	Lonoctocog alfa (Afstyla)	rFVIII single-chain	BDD-FVIII	24	50% (12/24)	25% (6/24)
PUPs A-LONG [30]	Open-label, multicenter, phase 3 study (NCT02234323)	Efmoroctocog alfa (Elocta/Eloctate)	Extended half-life FVIII	Full-length FVIII	101	31.1% (28/90^a^)	15.6% (14/90^a^)
Previously untreated young children [31]	Prospective, uncontrolled, open-label, multicenter study, phase 3 (NCT02615691)	Rurioctocog alfa pegol (Adynovi/Adynovate)	Extended half-life rFVIII	Full-length FVIII	59	19% (10/52)	9.6% (5/52)
Pathfinder6 [32]	International, single-arm, open-label, phase 3 trial (NCT02137850)	Turoctocog alfa pegol (Esperoct)	Extended half-life rFVIII	BDD-FVIII	81	30% (21/70)	15.7% (11/70)

BDD, B-domain deleted; EDs, exposure days; F, factor; PUP, previously untreated patient; rFVIII, recombinant factor VIII.

a Patients with ≥10 EDs.

On the whole, it is notable that all these observational studies may be affected by confounding by indication, meaning that the choice of FVIII product may be influenced by clinicians’ prior perceptions of inhibitor risk. However, the understanding of the real immunogenicity of novel rFVIII (Fc-fusion, PEGylated, and VWF D'-D3 domain-fusion) in the era of bispecific products requires further investigation.

### 3.2 Adoption in clinical practice

Adoption of the SIPPET findings in routine practice has been tempered by methodological and logistical constraints. In the United States, a survey of the Hemophilia and Thrombosis Research Society demonstrated that although 60% of clinicians expressed reservations about the SIPPET design, more than half of them shared the results with the families of newly diagnosed PUPs [33]. Furthermore, the use of rFVIII in PUPs decreased from 70% to 28%, while pdFVIII use increased, reflecting a gradual integration of the evidence stemming from SIPPET into clinical decision making [33]. Notably, post hoc SIPPET analyses emphasizing early immunogenicity prompted 70% of surveyed clinicians to preferentially use pdFVIII in PUPs [33]. Similar shifts have been observed in other countries. For instance, Italian hemophilia centers reported that following SIPPET there was an increased choice of pdFVIII in PUPS, reflecting the growing awareness of the inhibitor risk and the potential benefits of pdFVIII products [34]. In addition, the French Society of Hemophilia stated that, although SIPPET is unlikely to completely change clinical practice, its findings should prompt caution in the use of rFVIII in PUPs, particularly in those at high risk of inhibitor development [35]. To sum up, these real-world observations highlight the SIPPET influence beyond the original cohort, indicating a paradigm shift in PUP management.

### 3.3 Global impact and guideline evolution

SIPPET findings did impact worldwide and influenced clinical practice, guidelines, and patient-physician communications. The World Federation of Hemophilia, third edition, guidelines [36] explicitly mentioned SIPPET, stating that recombinant and plasma-derived products cannot be considered equivalent regarding inhibitor risk. While rFVIII remains standard choice in most high-income countries, the World Federation of Hemophilia guidelines recommend clinicians to discuss both options with patient families, emphasizing the differential immunogenicity.

In Europe, several national bodies, including Coordination Médicale pour l'étude et le traitement des maladies hémorragiques constitutionnelles (CoMETH) [35], the French Society of Hemophilia, issued advisories urging clinicians to consider SIPPET results during therapeutic decision making. The United States National Bleeding Disorders Foundation (formerly National Hemophilia Foundation) adopted a more cautious stance, acknowledging the findings but citing the predominance of rFVIII use and the emergence of novel nonfactor therapies [37].

The International Society on Thrombosis and Haemostasis (ISTH) clinical practice guideline for the management of congenital HA issued a conditional recommendation in favor of pdFVIII over standard plasma half-life rFVIII for PUPs with severe disease starting prophylaxis [38]. However, the implementation of this recommendation depends on product availability, costs, and patients’ preference and may be impractical in countries where pdFVIII is no longer considered standard of care. Moreover, the ISTH recommendation applies only to the pdFVIII products tested in the SIPPET trial and may not be generalizable to all FVIII products. The guideline also does not cover EHL rFVIII, a product class not evaluated in SIPPET. Nevertheless, a number of observational studies suggests that inhibitor incidence with EHL rFVIII is broadly comparable with that of standard half-life rFVIII (approximately 30%) (Table 2) [[30], [31], [32]].

While the current ISTH guideline [38] focuses on primary inhibitor prevention in PUPs, it is important to consider the challenges to be faced for management when patients develop a FVIII inhibitor while on prophylaxis. Despite recent advances in HA therapy, particularly with the introduction of nonreplacement agents such as emicizumab and rebalancing agents, FVIII inhibitors remains a major clinical challenge, particularly when patients with inhibitors on prophylaxis with nonreplacement agents bleed, spontaneously, owing to trauma or during invasive procedures. Indeed, the risk of inhibitor development has not been eliminated but rather shifted and extended over a longer period; as reported earlier, EUHASS registry data showed a significant decline in cumulative inhibitor incidence in severe HA from 24% in 2016 to 6% in 2022, coinciding with the increasing adoption of emicizumab prophylaxis in approximately 44% of PUPs [8].

### 3.4 Safety of switching products

The advent of EHL rFVIII products led a number of caregivers to favor them over pdFVIII for treating PUPs, so that concerns have been raised regarding the safety of switching from pdFVIII to rFVIII after initial exposure to pdFVIII. The Inhibitor development upon switching from plasma-derived to recombinant factor VIII in previously untreated patients with severe hemophilia A (PUP-SWITCH) study [39] specifically addressed this question, evaluating 87 PUPs switched from pdFVIII to rFVIII after the first 50 exposure days. Only 1 patient developed a novel inhibitor after switching, yielding a cumulative incidence of 1.15% (95% CI, 0.03%-6.24%), similar to that observed in previously treated patients [39]. These data suggest that switching after the early high-risk exposure window may be clinically safe and does not induce a secondary peak of immunogenicity when there is the need of switching products due to supply, availability, or patient preference.

To sum up, SIPPET and post-SIPPET evidence indicates that inhibitor development in PUPs results from an interplay among product characteristics, patient-related risk factors, and treatment strategies. While observational studies and registries have yielded partially varied results, real-world data reinforced the signal of lower immunogenicity associated with VWF-containing pdFVIII products and contributing worldwide to changes in clinician behavior, guideline recommendations, and patient counseling. At the same time, the emergence in the last 10 years of EHL rFVIII products and nonreplacement therapies has reshaped rather than eliminated the clinical relevance of inhibitors by shifting their timing and management challenges [8]. Taken together, these findings support a more individualized approach to the initial therapy selection in PUPs, balancing immunogenicity, safety, availability, and patient preference within an increasingly complex therapeutic landscape.

## 4 Mechanistic Insights: The Role of FVIII Immunogenicity, VWF, Genetic Factors, Epitope Profiling, and Epigenetics

**Figure 1:**
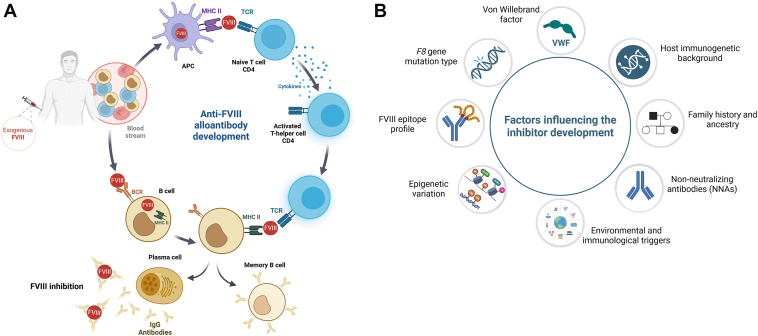
Mechanistic insights into factor (F)VIII immunogenicity. (A) Anti-FVIII alloantibody development. (B) Factors influencing inhibitor development. Created with BioRender.com. APC, antigen-presenting cell; BCR, B cell receptor; IgG, immunoglobulin G; MHC, major histocompatibility complex; TCR, T cell receptor; VWF, von Willebrand factor.

During early life, the immune system establishes self-tolerance primarily through central tolerance, a process that eliminates or inactivates T and B lymphocytes reactive to self-antigens (Figure 1). However, in patients with severe HA, FVIII is not endogenously expressed and is perceived as foreign [40]. Thus, when FVIII is administered exogenously, it is recognized by the immune system as nonself and is taken up by dendritic cells via receptor-mediated endocytosis, thus leading to antibody formation (Figure 1A). FVIII immunogenicity is governed by a complex interplay of protein-level, immunologic, genetic, and epigenetic factors (Figure 1B). In addition, studies arising from SIPPET cohort provided the opportunity to validate and integrate these mechanisms within a prospective clinical framework.

### 4.1 Role of VWF

Mechanistic insights suggest that VWF contained in pdFVIII plays a critical immunomodulatory role. When FVIII is bound to VWF as in pdFVIII products, VWF shields FVIII from uptake by antigen-presenting cells, quenching its presentation to the immune system. Accordingly, the FVIII–VWF complex may be less efficiently presented to developing T cells, thereby limiting the induction of central tolerance. Additionally, VWF competes with inhibitory antibodies for binding to the C2 domain of FVIII, thus potentially reducing the functional impact of low-titer antibodies. Hartholt et al. [41] further demonstrated that the presence of VWF–FVIII complexes on dendritic cell surfaces modulates internalization pathways and that the incomplete sulfation of Tyr1699 in recombinant products reduces VWF binding and potentially increases immunogenicity. These observations provide biological plausibility to the protective effect of VWF-containing pdFVIII products observed in SIPPET. Further evidence supporting that VWF may reduce FVIII immunogenicity is provided by the recently available efanesoctocog alfa, an rFVIII complex molecule incorporating not only the VWF D'D3 domain but also XTEN polypeptides and T cell regulatory epitopes. This product is postulated to thwart the development of a T cell–mediated neutralizing immune response and thus potentially reduce the risk of neutralizing antibodies, particularly in PUPs [42]. However, more data on its immunogenicity in PUPs are required.

### 4.2 Role of genetics

Genetic factors remain among the strongest predictors of inhibitors. Mutations in the *F8* gene are classified as null or nonnull based on the predicted residual synthesis of FVIII. Null mutations, such as large deletions, nonsense variants, and intron 22 inversions, confer a higher risk, whereas nonnull mutations (including certain missense and splicing variants) allow residual protein production that might promote immune tolerance to FVIII [43].

Rosendaal et al. [44] performed a stratified analysis within the SIPPET cohort, demonstrating that patients with null mutations had a 31% cumulative incidence of inhibitors even when treated with pdFVIII, whereas those with nonnull mutations exhibited a negligible inhibitor risk with this source of therapy. Notably, the incidence of inhibitors among low- and high-risk cases treated with rFVIII did not differ substantially (43% vs 47%), indicating that recombinant products may override the putative protective effect of residual FVIII synthesis.

Spena et al. [45] further refined genetic risk assessment within the SIPPET cohort by combining *F8* mutational analysis with FVIII antigen measurements and showed a 2-fold increased risk of inhibitor for *in silico* null mutations vs nonnull mutations and a 3.5-fold increase in antigen-negative vs antigen-positive patients, confirming that residual FVIII antigen induces immune tolerance. These results underscore the usefulness of combining mutation type and FVIII antigen levels for predicting inhibitor risk and thus tailoring therapy.

Family history of inhibitors is another heritable risk factor, potentially reflecting shared immune-regulatory polymorphisms [46]. Specific human leukocyte antigen haplotypes and other immune gene variants have been implicated, but their predictive value is limited in clinical practice, so that the integration of mutation type, antigen expression, and family history remains the most robust strategy for risk assessment [46].

### 4.3 Nonneutralizing antibodies and immunological mechanisms

Nonneutralizing anti-FVIII antibodies (NNAs) are detectable in a few patients with HA before treatment and have been associated to an increased inhibitor risk [47]. In SIPPET, plasma samples collected before any exposure to FVIII were analyzed to determine the prevalence of NNAs and evaluate the association with inhibitor onset; 7.6% of patients had NNAs at baseline, and their presence conferred an 83% higher risk of inhibitor, high-titer inhibitors showing almost a 3-fold increase [47]. NNAs may act as the priming signal that the immune system recognized FVIII as a foreign antigen and thus facilitate the activation of antigen-specific B cells upon FVIII exposure.

### 4.4 IgG subclasses and epitope recognition

Longitudinal studies of IgG subclasses in SIPPET revealed that the number and type of anti-FVIII IgG subclasses correlate with inhibitor persistence [48]. Anti-FVIII IgG2 emerged as a hallmark of persistent inhibitors over a 6-month follow-up period, and the concomitant presence of multiple IgG subclasses during early inhibitor development was associated with higher persistence [48]. These immunologic biomarkers may help to distinguish transient from persistent inhibitors, providing potential for prognostic stratification and tailored therapeutic planning. Thus, monitoring IgG subclass profiles may provide insights into inhibitor evolution and persistence and guide future strategies for immune tolerance induction or novel immunomodulatory approaches.

Epitope-level profiling added a new dimension to predicting inhibitor risk. Hassan et al. [49] used a random peptide phage-display assay in 122 PUPs from the SIPPET cohort with the goal to characterize FVIII-specific IgG epitopes before and after FVIII exposure. The study identified 27,775 peptides potentially directed against FVIII and used least absolute shrinkage and selection operator regression and random forest models to predict inhibitor development. Both models demonstrated good discrimination (*C*-statistics, 0.78 and 0.80) and moderate calibration, suggesting that epitope-specific profiling may help to stratify patients for their immunologic risk prior to the first FVIII exposure.

### 4.5 Epigenetic contributions

Recent research explored the contribution of epigenetic mechanisms. Chand et al. [50] investigated DNA methylation in peripheral blood mononuclear cells from inhibitor-positive and -negative SIPPET PUPs, analyzing over 600,000 CpG sites. They identified 2772 differentially methylated sites associated with genes involved in immune regulation, including *JAK1*, *CD1C*, *TOLLIP*, *BLNK*, and *IL23R* [50]. These findings do not identify a specific mechanistic pathway but suggest that epigenetic variation may modulate individual susceptibility to FVIII immunogenicity and demand further investigation. Integrating genomics, epigenetics, and immunophenotyping might enable more precise stratification and optimized therapeutic strategies.

Together, many of these mechanistic insights, including VWF-mediated protection, NNAs, *F8* genotype, epitope specificity, and epigenetic regulation, were further refined and clinically contextualized by analyses carried out in the frame of the SIPPET cohort. They provide a comprehensive framework for better understanding the mechanism of FVIII immunogenicity. They also lay the foundation for predictive and personalized approaches in hemophilia care, particularly at the current era of nonreplacement therapies and thus at a time when the achievement of tolerization to FVIII might require a longer period of time, with the added risk of loss of peripheral tolerization owing to the scarcity of FVIII exposure.

## 5 Integrating Mechanistic and Clinical Insights into Clinical Strategy

The combination of mechanistic understanding and clinical evidence should allow for a more nuanced and patient-centered approach. However, attempts to predict inhibitor development in severe HA have so far yielded modest results. Hassan et al. [51], by analyzing the SIPPET cohort, attempted a rigorous external validation of a previously proposed model incorporating family history, *F8* mutation type, and treatment intensity [52]. The model performed poorly (*C*-statistic, 0.53), with little discriminatory capacity and limited calibration, in contrast with previous findings [52] that these variables were able to stratify risk. Hassan et al. [51] highlight the difficulty of replicating accuracy, particularly across diverse patient populations, and underscore the need for more comprehensive models incorporating additional immunogenetic and treatment-related factors. They further proposed a new 4-variable model, integrating FVIII product type and the presence of nonneutralizing antibodies prior to treatment initiation. While this refinement improved discrimination slightly (*C*-statistic, 0.66), clinical utility was essentially restricted to rule out a high inhibitor risk, given its high negative predictive value (0.95 at the 10% threshold) but limited positive predictive capacity [51]. This study underscores a broader limitation of current prediction research, that is, reliance on relatively few clinical and genetic variables. Although useful for exploratory modeling, these variables are insufficient to capture the multifactorial nature of inhibitor development, which is likely to involve complex immunogenetic interactions and treatment-related triggers. As others have argued [[53], [54], [55]], more promising avenues may stem from the integration of immunological biomarkers, high-resolution genetic data, or machine learning frameworks capable such a high degree of complexity. Accordingly, the work by Hassan et al. [51] is valuable not for providing a definitive tool but for more clearly illustrating the gap between current predictive efforts and the requirements of truly precise medicine in hemophilia.

## 6 Conclusion and Future Directions

The SIPPET trial and related studies have unequivocally demonstrated that pdFVIII containing VWF carries a lower risk of inhibitor development than rFVIII in PUPs and that the earliest exposure days identify a window of heightened immunological vulnerability. Genetic, immunologic, and perhaps epigenetic factors further modulate individual susceptibility, reinforcing the need for early risk stratification and informed product selection.

All in all, inhibitor development remains a multifactorial, dynamic process that currently available predictive models capture only partially. Enhancing predictive models through high-resolution immunogenomic and epigenomic profiling is a promising avenue in order to improve risk stratification and tailor therapy more precisely.

The advent of nonreplacement therapies, such as emicizumab, newly emerging bispecific antibodies, and rebalancing agents, presents both opportunities and challenges. Even though these agents reduce bleeding rates, delay FVIII exposure and potentially mitigate inhibitor risk, they do not replace the need for FVIII in acute or surgical settings. In this evolving landscape, artificial intelligence (AI) and machine learning frameworks offer unprecedented opportunities to integrate complex high-dimensional datasets, including genetic, immunologic, epigenetic, and clinical variables, into robust predictive algorithms capable of supporting individualized therapeutic decisions, as recently demonstrated by machine learning models that successfully predicted inhibitor development in previously untreated and minimally treated children with hemophilia [56].

Looking forward, hemophilia is poised to move toward truly personalized care. Priorities include refining mechanistic understanding in order to identify actionable immunomodulatory targets, enhance predictive models through high-resolution immunogenomic and epigenomic profiling, develop AI-driven risk stratification tools, and evaluate the real-world impact of evolving therapies on inhibitor incidence and healthcare costs. International collaboration and large-scale longitudinal studies are essential to validate these approaches across diverse patient populations.

In conclusion, 10 years ago, the SIPPET trial laid a critical foundation, providing high-quality evidence to guide initial therapy decisions and highlighting the pivotal role of early FVIII exposure. At the same time, its limitations emphasize that the next era of care of PUPs with severe hemophilia will depend on integrating mechanistic insights, advanced predictive tools, AI-assisted modeling, and innovative therapeutic strategies into a flexible, patient-centered framework moving closer to precision medicine.
